# The relationship between sensory characteristics and emotional/behavioral problems in preschool children-the moderating role of maternal parenting stress

**DOI:** 10.3389/fpsyg.2025.1683823

**Published:** 2025-12-10

**Authors:** Zhanbin Xu, Hongchao Qin, Licheng Shi, Yong Ni, Qiuchan Qu, Xiangtian Kong, Feiying Wang

**Affiliations:** 1 Department of Child Healthcare, Affiliated Maternity and Child Health Care Hospital of Nantong University, Nantong, Jiangsu, China; 2 Department of Child and Adolescent Health, Public Health College, Harbin Medical University, Harbin, Heilongjiang, China; 3 Child Health Department of Rudong County Maternal and Child Health and Family Planning Service Center, Nantong, Jiangsu, China; 4 Department of Psychology, Nantong University, Nantong, Jiangsu, China; 5 Department of Medical Genetics and Prenatal Diagnosis, Affiliated Maternity and Child Health Care Hospital of Nantong University, Nantong, Jiangsu, China

**Keywords:** sensory characteristics, emotional and behavioral problems, preschool children, parenting stress, moderating effect

## Abstract

**Object:**

To examine the relationship between sensory characteristics and emotional/behavioral problems in preschool children, and to explore the moderating role of maternal parenting stress.

**Method:**

From April to December 2024, a total of 321 healthy children and their primary caregivers from six kindergartens in Nantong City were recruited as study participants. The Short Sensory Profile, Strengths and Difficulties Questionnaire, and Parenting Stress Index-Short Form were used to assess children’s sensory characteristics, emotional and behavioral problems, and maternal parenting stress.

**Result:**

The average total score of the SSP among the 321 preschool children was 57.09 ± 16.59, with an abnormality rate of 5.6%. Among the domains of the SDQ, peer relationship problems had the highest rate of abnormalities (31.2%), followed by hyperactivity/inattention (7.79%), emotional symptoms (5.30%), total difficulties (4.36%), conduct problems (3.74%), and prosocial behavior (3.43%). The total SSP score was correlated with all dimensions of the SDQ (*P* < 0.01), and maternal parenting stress was correlated with all dimensions of the SDQ (*P* < 0.05). Sensory features demonstrated a significant negative predictive effect on emotional and behavioral problems (β = −0.36, *P* < 0.01), while parenting stress showed a significant positive predictive effect on emotional and behavioral problems (β = 0.27, *P* < 0.01). Additionally, the interaction term between sensory features and parenting stress significantly and negatively predicted emotional and behavioral problems (β = −0.15, *P* < 0.01). When maternal parenting stress was high (+1 SD), children’s sensory abnormalities had a strong positive influence on emotional and behavioral problems (*t* = 9.07, *P* < 0.001). Conversely, when maternal parenting stress was low (−1 SD), the predictive significance of children’s sensory abnormalities on emotional and behavioral problems was weaker (*t* = 3.17, *P* < 0.01).

**Conclusion:**

There was a significant association between sensory characteristics and emotional/behavioral problems in preschool children. Maternal parenting stress moderated this relationship—higher levels of parenting stress amplified the impact of children’s sensory characteristics on emotional and behavioral issues. Future efforts should place greater emphasis on the development of sensory characteristics in preschool children and address family-related parenting stress, in order to effectively reduce the occurrence of emotional and behavioral problems in early childhood.

## Introduction

Emotional and behavioral problems (EBPs) in children refer to emotional responses and behavioral expressions that exceed the normative expectations for their age group. It has been reported that the occurrence and development of EBPs are influenced by various environmental factors, including maternal parenting stress, emotional disorders in siblings, excessive use of social media, and academic-related stress (Fang et al., 2024; Smorti et al., 2021; Wang et al., 2025; Yu et al., 2025). Recent studies have further suggested that EBPs in children with neurodevelopmental disorders are associated with sensory abnormalities (Chen et al., 2022; Fabbri-Destro et al., 2022), Currently, there is a relative scarcity of research on the relationship between sensory characteristics in typically developing children and their emotional/behavioral problems, as well as maternal parenting stress.

Emotional and behavioral problems during childhood can have far-reaching consequences, potentially affecting academic achievement, social adaptation, peer interactions, family dynamics, and the parent–child relationship. Moreover, these adverse effects often persist into adulthood (Krause et al., 2023). Therefore, the prevention and intervention of EBPs in preschool children have become a key focus in current public health research.

This study aims to explore the relationship between sensory characteristics and emotional/behavioral problems in preschool children, as well as the moderating role of maternal parenting stress, in order to provide both theoretical and practical guidance for the early screening and intervention of children’s mental health issues.

## Subjects and methods

### Study subjects

A stratified sampling method was employed to enroll 321 healthy children and their primary caregivers from two kindergartens in each of three representative districts (with good, moderate, and poor economic development levels) in Nantong City between April 2024 and December 2024 as study subjects. Among the participants, 154 were boys (47.98%) and 167 were girls (52.02%), with an average age of 4.83 years (SD = 0.799). The study was approved by the Ethics Committee of Nantong Maternal and Child Health Hospital (Ethics Approval No.: Y2024002), and informed consent was obtained from all primary caregivers.

### Study methods

#### Parenting Stress Index-Short Form (PSI-SF)

The PSI-SF (Luo et al., 2021) is a widely used tool for evaluating parenting stress. It consists of 36 items scored on a five-point Likert scale (1 = strongly disagree to 5 = strongly agree). Higher scores indicate greater parenting stress. Total scores range from 36 to 180, with a score of 90 defined as the critical threshold. Scores above 90 indicate high levels of parenting stress, while scores of 90 or below suggest lower levels of stress. Parenting stress was categorized into four levels: normal (≤ 85), borderline high (86–90), high (91–98), and very high (≥ 99). This scale has been widely used internationally and in China (Amaerjiang et al., 2021; Cazzorla et al., 2025), demonstrating good reliability and validity in the Chinese context. In this study, Cronbach’s α coefficient was 0.935, RMSEA was 0.03, and CFI was 0.975.

#### Strengths and Difficulties Questionnaire (SDQ)

The SDQ (Goodman, 1997) is commonly used to assess children’s emotional and behavioral problems, with ratings provided by the primary caregiver based on the child’s behavior over the past six months. The scale includes 25 items rated on a three-point scale (0 = not true, 1 = somewhat true, 2 = certainly true), Items 7, 11, 14, 21, and 25 are reverse-scored, thereby assessing five factors: emotional symptoms, conduct problems, hyperactivity/inattention, peer relationship problems, and prosocial behavior, along with a total difficulties score. The SDQ cutoff criteria are based on Chinese population norms. For emotional symptoms, scores of 0–5 are normal, 6 is borderline, and 7–10 are abnormal. For conduct problems, scores of 0–3 are normal, 4 is borderline, and 5–10 are abnormal. For hyperactivity/inattention, scores of 0–5 are normal, 6 is borderline, and 7–10 are abnormal. For peer relationship problems, scores of 0–3 are normal, 4–5 are borderline, and 6–10 are abnormal. For prosocial behavior, scores of 6–10 are normal, 5 is borderline, and 0–4 are abnormal. For total difficulties, scores of 1–15 are normal, 16–19 are borderline, and 20–40 are abnormal. Higher scores in emotional symptoms, conduct problems, hyperactivity/inattention, peer relationship problems, and total difficulties, as well as lower scores in prosocial behavior, indicate more severe emotional and behavioral problems. This scale has been widely used internationally and in China (Deng et al., 2023; Ribeiro Santiago et al., 2022), demonstrating good reliability and validity. In this study, the Cronbach’s α coefficient was 0.776, RMSEA was 0.04, and CFI was 0.951.

#### Short Sensory Profile (SSP)

The SSP (Mcintosh et al., 1999) is one of the most widely used questionnaires for assessing sensory characteristics in children aged 3–10, particularly for screening sensory processing abnormalities. It covers eight dimensions: tactile sensitivity, taste/smell sensitivity, movement sensitivity, underresponsive/seeks sensation, auditory filtering, low energy/weak, visual/auditory sensitivity, and total score, comprising 38 items. Items are rated on a 5-point scale based on the frequency of specific behaviors. Scores in each dimension are categorized into three levels: typical performance, probable difference, and definite difference. The criteria for defining abnormal values in SSP are based on international population standards. This scale is widely used internationally and in China (Lyons-Warren et al., 2022; Zhai et al., 2023), demonstrating good reliability and validity in the Chinese context. The SSP total scores are interpreted as follows: 155–190 indicates basic normality, 142–154 suggests possible abnormality, and 38–141 indicates marked abnormality, with lower scores suggesting more pronounced sensory abnormalities. In this study, the Cronbach’s α coefficient was 0.910, RMSEA was 0.03, and CFI was 0.986.

### Quality control

All researchers involved in the study received standardized training. Caregivers completed the questionnaires under the guidance of trained researchers. On-site quality control was conducted during questionnaire completion. Data were collected, organized, and analyzed by designated personnel, and all data were double-checked to ensure accuracy and reliability.

### Data analysis

Statistical analysis was conducted using SPSS version 23.0. Descriptive statistics for continuous variables were presented as mean ± standard deviation ($\bar{x}$ ± s). Independent-samples *t*-tests were used to compare means between two groups. Chi-square (χ^2^) tests were used for comparing proportions. The moderation effect was tested using the PROCESS macro developed by Hayes, with the significance level set at α = 0.05.

## Results

### Distribution of sensory characteristics, emotional and behavioral problems, and maternal parenting stress in preschool children

The results showed that the average total score on the SSP among 321 preschool children was 57.09 ± 16.59, with an abnormal rate of 5.61% for overall sensory characteristics. Among the SDQ subscales, peer relationship problems had the highest abnormal rate (31.15%), followed by hyperactivity/inattention (7.79%), emotional symptoms (5.30%), total difficulties (4.36%), conduct problems (3.74%), and prosocial behavior (3.43%). The average maternal parenting stress score was 75.06 ± 18.01, with an abnormal rate of 28.04%. Detailed results are shown in Table 1.

**TABLE 1:** Distribution of sensory characteristics, emotional and behavioral problems, and maternal parenting stress in preschool children *n* (%).

Variant	Sensory characteristics	Emotional symptoms	Conduct problems	Hyperactivity and inattention	Peer relationship problems	Prosocial behavior	Total difficulties	Parenting stress
Mean (M)	57.09	1.60	1.45	3.81	3.01	7.80	9.88	75.06
Standard deviation (SD)	16.59	1.46	1.04	1.75	1.14	1.81	3.46	18.01
Minimum	35.0	0	0	1.0	0	2.0	3.0	40.0
Maximum	118.0	8.0	5.0	9.0	6.0	10.0	24.0	125.0
Abnormal (*n*, %)	18 (5.61)	17 (5.30)	12 (3.74)	25 (7.79)	100 (31.15)	11 (3.43)	14 (4.36)	90 (28.04)

### Correlation between sensory characteristics, emotional and behavioral problems, and maternal parenting stress

The total SSP score of preschool children was negatively correlated with SDQ subscales including emotional symptoms, conduct problems, hyperactivity/inattention, peer relationship problems, and total difficulties (*r* = −0.41, −0.35, −0.39, −0.25, −0.56; all *P* < 0.01). It was positively correlated with prosocial behavior (*r* = 0.39, *P* < 0.01).

Maternal parenting stress was positively correlated with children’s emotional symptoms, conduct problems, hyperactivity/inattention, peer relationship problems, and total difficulties (*r* = 0.38, 0.30, 0.35, 0.18, 0.47; all *P* < 0.05), and negatively correlated with prosocial behavior (*r* = −0.43, *P* < 0.01). Detailed results are shown in Table 2.

**TABLE 2:** Correlation between the total score of SSP and emotional behavioral problems in preschool children.

Variant	1. Sensory characteristics	2. Emotional symptoms	3. Conduct problems	4. Hyperactivity and inattention	5. Peer relationship problems	6. Prosocial behavior	7. Total difficulties	8. Parenting stress
1. Sensory characteristics	1							
2. Emotional symptoms	−0.41**	1						
3. Conduct problems	−0.35**	0.27**	1					
4. Hyperactivity and inattention	−0.39**	0.20**	0.29**	1				
5. Peer relationship problems	−0.25**	0.16**	0.14*	0.026	1			
6. Prosocial behavior	0.39**	−0.31**	−0.40**	−0.27**	−0.29**	1		
7. Total difficulties	−0.56**	0.64**	0.61**	0.69**	0.451*	−0.48**	1	
8. Parenting stress	−0.47**	0.38**	0.30**	0.35**	0.184*	−0.43**	0.47**	1

**Correlation is significant at the 0.01 level (two-tailed);

*Correlation is significant at the 0.05 level (two-tailed).

### Regression analysis of sensory characteristics, emotional and behavioral problems, and maternal parenting stress

A hierarchical regression analysis was conducted to examine the moderating role of maternal parenting stress in the relationship between children’s sensory characteristics and emotional/behavioral problems. Considering that demographic variables and family socioeconomic factors, among others, may act as confounders in the model, the first layer of the hierarchical regression analysis incorporated control variables including gender, grade level, number of children, family structure, and parental education and employment status. In the second step, the centered total scores of sensory characteristics and parenting stress were added. In the third step, the interaction term between sensory characteristics and parenting stress was included to test the moderating effect.

The results showed that sensory characteristics significantly negatively predicted emotional and behavioral problems (β = −0.36, *P* < 0.01), and maternal parenting stress had a significant positive predictive effect (β = 0.27, *P* < 0.01). Additionally, the interaction term between sensory characteristics and maternal parenting stress significantly negatively predicted emotional and behavioral problems (β = −0.15, *P* < 0.01). This indicates that maternal parenting stress plays a moderating role in the relationship between children’s sensory characteristics and their emotional/behavioral problems (see Table 3).

**TABLE 3:** Regression analysis of sensory characteristics, emotional and behavioral problems, and maternal parenting pressure in preschool children.

Variant		Model 1		Model 2		Model 3	
		β	*t*	β	*t*	β	*t*
Control variant	Gender	−0.07	−1.14	−0.02	−0.15	−0.01	−0.09
	Age	−0.09	−3.06**	−0.08	−2.84**	−0.06	−2.28*
	Grade	−0.05	−1.13	−0.03	−0.79	−0.02	−0.40
	Number of children	−0.11	−3.30**	−0.09	−2.78**	−0.06	−2.31*
	Family structure	−0.07	−4.91***	−0.05	−2.85**	−0.04	−2.79**
	Mother’s educational level	−0.12	−4.22***	−0.11	−4.01***	−0.09	−3.76***
	Father’s educational level	−0.08	−0.95	−0.06	−0.82	−0.05	−0.77
	Mother’s employment status	−0.10	−4.44***	−0.9	−4.02***	−0.07	−3.89***
	Father’s employment status	−0.09	−1.27	−0.06	−0.99	−0.05	−0.56
Independent variant	Sensory characteristics			−0.40	−7.36***	−0.36	−6.64***
Moderator variant	Parenting stress			0.29	5.33***	0.27	5.88***
Interaction	Sensory characteristics * parenting stress					−0.15	−3.40**
	R^2^	0.07		0.42		0.45	
	ΔR^2^	0.06		0.39		0.42	
	*F*	3.57*		21.33***		25.96***	

*P < 0.05;

**P < 0.01;

***P < 0.001.

**FIGURE 1:**
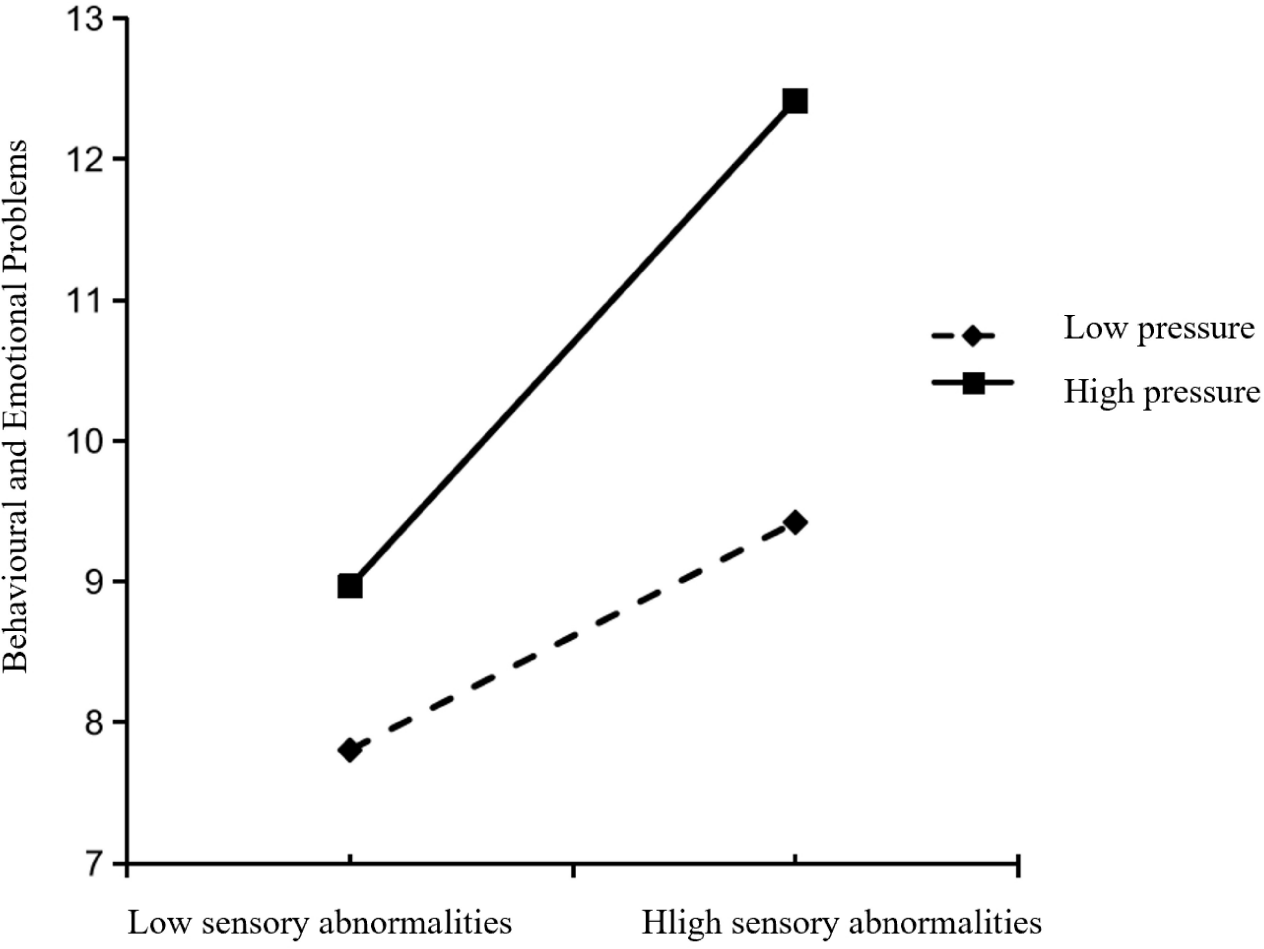
Simple slope test of maternal parenting stress moderation effect.

**FIGURE 2:**
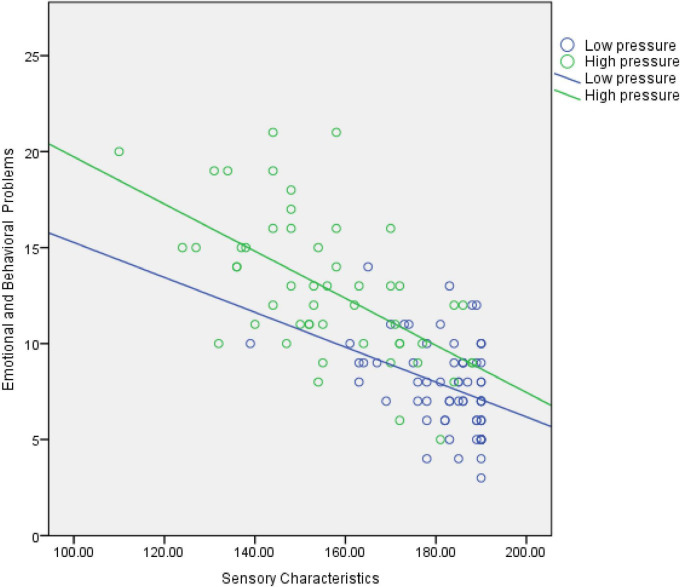
Scatter plot of maternal parenting stress moderation effect. Low stress R^2^ = 0.147, high stress R^2^ = 0.347.

To more clearly illustrate the moderating effect of maternal parenting stress on the pathway between children’s sensory features and emotional/behavioral problems, parenting stress was divided into high and low level groups based on one standard deviation above and below the mean. Simple slope tests were conducted and scatter plots were generated to show the association between sensory features and emotional/behavioral problems. The results indicated that when maternal parenting stress was high (+1 SD), the positive effect of sensory abnormalities on emotional and behavioral problems was stronger (*t* = 9.07, *P* < 0.001). When maternal parenting stress was low (−1 SD), the predictive effect was weaker but still significant (*t* = 3.17, *P* < 0.01), in Figure 1; The scatter plot results indicate that emotional and behavioral problems in both the high-stress and low-stress groups decrease as sensory feature scores increase, with a more pronounced downward trend in the high-stress group. Under the same sensory feature conditions, children in the high-stress group exhibit more severe emotional and behavioral problems, in Figure 2.

## Discussion

The preschool stage represents a critical developmental window in a child’s growth trajectory. During this period, children experience significant physical development as well as deep psychological changes, with sensory characteristics and emotional and behavioral patterns beginning to emerge. Studies have shown that early sensory development has a profound impact on cognition, emotion, and social skills, and is associated with the occurrence of emotional and behavioral problems in children (Chen et al., 2024). Moreover, research suggests that maternal parenting stress is related to the emergence of children’s emotional and behavioral problems and often plays an important mediating and moderating role in family relationships and negative health outcomes (Chung et al., 2022; Kochanova et al., 2022; Liu et al., 2024). Therefore, this study aimed to explore the relationship between preschool children’s sensory characteristics and emotional/behavioral problems, as well as the moderating role of maternal parenting stress, with the goal of promoting early prevention and intervention and supporting children’s healthy development.

Children explore and understand the world through their senses. Sensory characteristics play a crucial role in their development, forming the foundation for cognitive, motor, and social abilities. This study found that out of 321 preschool children, 18 (5.61%) had abnormal sensory characteristics. This indicates that the majority of preschool children have typical sensory development without significant processing issues. However, the SSP’s broad definition of “normal” scores suggests that even within the normal range, there may be substantial individual differences in sensory processing. These findings highlight the importance of personalized attention and support from parents and educators. Currently, there is a lack of research on abnormal sensory characteristics in preschool-aged children, whereas studies focusing on children with neurodevelopmental disorders—such as autism—report sensory abnormalities in approximately 74.0% to 92.10% of cases (Kirby et al., 2022; Posar and Visconti, 2018; Section on Complementary and Integrative Medicine et al., 2012). This underscores the close relationship between sensory features and psychological and behavioral development. Sensory abnormalities are likely associated with developmental and behavioral disorders and therefore warrant closer monitoring in preschool-aged children. There is still a lack of research on the mechanisms and interventions for sensory abnormalities in children (Bang et al., 2024; Unwin et al., 2022), and further studies are needed to clarify the causes and influencing factors of sensory differences.

Compared to children in other age groups, preschoolers exhibit a wide range of emotional and behavioral problems. These issues not only include observable behaviors such as irritability, excessive screen time, and social withdrawal, but also more covert symptoms such as anxiety, depression, and obsessive-compulsive tendencies, which are often difficult for parents to detect. Multiple factors influence the development of emotional and behavioral problems in preschool children. For example, Martinsone et al. (2021) found that socialization skills in preschoolers are related to emotional and behavioral issues, and that early support for social and emotional competencies is key to academic and behavioral success. Research in China indicates that adverse childhood experiences in mothers can increase the risk of emotional and behavioral problems in their preschool-aged offspring. Wang et al. (2022); Cooke et al. (2019) showed that parents with better emotional regulation tend to exhibit more positive parenting behaviors, and their children in turn show better emotional regulation and fewer internalizing symptoms (e.g., anxiety, depression). Additional contributing factors include social stimuli, sibling emotional/social difficulties, excessive social media use, and academic pressure (Boer et al., 2020; Hobby et al., 2023; Li et al., 2022).

This study found that peer relationship problems were the most common emotional and behavioral issue among preschoolers (31.15%), Other emotional and behavioral issues—such as hyperactivity/inattention, emotional symptoms, total difficulties, conduct problems, and prosocial behavior—had relatively lower abnormal rates. Most preschool children in China are only children and often live in extended families with grandparents, which can lead to overindulgent parenting. In addition, the transition into group learning environments like kindergarten requires children to develop peer interaction skills and adapt to collective life, contributing to the high proportion of peer relationship problems indicated by the SDQ. Research also shows that gender and parenting style influence children’s emotional and behavioral development. Boys are more likely to exhibit hyperactivity and attention problems, while girls tend to show better social adaptation skills (Hinshaw et al., 2022; Yang et al., 2019). These findings suggest that parents and educators should pay greater attention to the emotional and behavioral health of boys in particular.

Furthermore, this study found that children’s total sensory scores were negatively correlated with emotional symptoms, conduct problems, hyperactivity/inattention, peer relationship problems, and total difficulties, and positively correlated with prosocial behavior. While, maternal parenting stress was positively correlated with all SDQ subscales except prosocial behavior, which was negatively correlated. These results suggest that children’s sensory characteristics significantly and negatively predict emotional and behavioral problems, while parenting stress showed a significant positive predictive effect on emotional and behavioral problems. Moreover, the interaction term between sensory features and parenting stress significantly and negatively predicted emotional and behavioral problems, indicating a moderating effect. When maternal parenting stress is high, the impact of children’s sensory abnormalities on emotional and behavioral problems is stronger; when maternal stress is low, this predictive relationship is weaker. Studies have shown that children with sensory abnormalities often exhibit more emotional and behavioral problems (Mulligan et al., 2021). These issues in children frequently exacerbate parenting stress, which in turn indirectly influences children’s emotional and behavioral problems by affecting parents’ harsh discipline practices (Jiang et al., 2023; Li et al., 2023; Zou, 2021). Additionally, the lack of social and policy support can increase anxiety, depressive symptoms, and parenting stress among parents of these children (Fang et al., 2024). Current research on the association between sensory features in typically developing children and parenting stress remains scarce. Studies on children with ASD have found that early sensory abnormalities can predict emotional and behavioral problems, thereby increasing parenting stress (Ben-Sasson et al., 2013; Fabbri-Destro et al., 2022). These findings underscore the importance of understanding and addressing both sensory development and maternal stress during early childhood, to facilitate early intervention and reduce the risk of emotional and behavioral problems in both childhood and later life.

In conclusion, sensory development in preschool children generally falls within the normal range, with only a minority exhibiting sensory abnormalities. However, emotional and behavioral problems increase with the severity of sensory abnormalities, showing a positive correlation. Maternal parenting stress moderates the relationship between children’s sensory characteristics and emotional/behavioral problems, with higher stress levels strengthening this relationship. The findings of this study can be applied to clinical practice, suggesting that greater attention should be paid to the development of children’s sensory characteristics in future diagnostic, therapeutic, and research work. It is essential to promptly assess maternal parenting stress and implement early proactive interventions for children with sensory processing abnormalities. Additionally, relevant departments should formulate supportive policies to reduce maternal parenting stress, thereby actively preventing and minimizing the occurrence of sensory processing abnormalities and emotional/behavioral problems in children, ultimately promoting their healthy overall development.

Limitations of this study: The samples in this study were all from Nantong City, which imposes certain limitations on the generalizability of the findings. The results cannot yet be extrapolated to national or international contexts. Future research should aim to expand the sample scope to obtain more representative results. This study is a cross-sectional survey and cannot establish causal relationships among the research variables. Additionally, all questionnaires involved were completed by the children’s mothers, which may inevitably introduce parental reporting bias. Subsequent studies should consider longitudinal research approaches, incorporating more potential variables (such as health status, paternal parenting styles, socioeconomic factors, etc.) and reports from teachers and clinicians to derive more accurate conclusions.

## Data Availability

The original contributions presented in this study are included in this article/supplementary material, further inquiries can be directed to the corresponding authors.
